# Comparative study on school-based mental health literacy in three Asian countries

**DOI:** 10.1186/s41182-025-00697-6

**Published:** 2025-06-23

**Authors:** Fumiko Shibuya, Masahide Usami, Marian Danille Santillan, Cut Warnaini, Ernesto Gregorio, Naoko Satake, Crystal Amiel Estrada, Gunawan Gunawan, Norieta Balderrama, Japhet Fernandez de Leon, Joie Fe Ancheta, Hamsu Kadriyan, Fernando Garcia, Jun Kobayashi

**Affiliations:** 1 Department of Global Health, Graduate School of Health Sciences, University of the Ryukyus, 1076 Kiyuna, Ginowan, Okinawa, 901-2725 Japan; 2 Japanese Consortium for Global School Health Research, Ginowan, Japan; 3 Department of Child and Adolescent Psychiatry, National Kohnodai Medical Center, Japan Institute for Health Security, 1-7-1 Kohnodai, Ichikawa, Chiba 272-8516 Japan; 4 Department of Health Promotion and Education, College of Public, Health University of the Philippines Manila, Manila, Philippines; 5 Faculty of Medicine, University of Mataram, Lombok, Indonesia; 6 Department of Psychiatry, National Kohnodai Medical Center, Japan Institute for Health Security, Ichikawa, Japan; 7 Department of Environmental and Occupational Health, College of Public, Health University of the Philippines Manila, Manila, Philippines; 8 Faculty of Teacher Training and Education, University of Mataram, Lombok, Indonesia; 9 College of Medicine, University of the Philippines Manila, Manila, Philippines; 10 Philippine Society of Child and Adolescent Psychiatry, Manila, Philippines; 11 West Visayas State University Medical Center, Iloilo City, Philippines; 12 Schools Division Office Manila, Department of Education, Manila, Philippines; 13 Department of Health Policy and Administration, College of Public Health, University of the Philippines Manila, Manila, Philippines

**Keywords:** Comparative study, Mental health literacy, Schools, Policy, Curriculum, Asia

## Abstract

**Introduction:**

Mental health literacy is essential for the recognition, management, and prevention of mental disorders among school-aged children. However, few studies have examined the implementation status of school-based mental health literacy in Asian countries. This study aims to compare the approaches taken by the Philippines, Indonesia, and Japan in managing school-based mental health literacy through curriculum-related policies.

**Methods:**

The collected documents of this study analyzed both policies (formulated from 2000 to 2023) and curricula (from grade 1 to grade 12) that were adapted to the deductive content analysis methods. Policies were analyzed using the policy triangle framework (Walt and Gilson in Health Policy Plan 9:353–370, 1994) and mapped using the review points (Margaretha et al. in Front Psychiatry 14:1126767, 2023). Curricula were analyzed using the definition of mental health literacy (Jorm in Am Psychol 67:231–243, 2012). This study focused on mental health laws and policies in the Philippines, Indonesia, and Japan, highlighting their success in addressing the needs of adults and school-aged children. By considering each country’s unique socio-cultural contexts and basic educational approaches, this study identified diverse strategies and methodologies in addressing mental health challenges. Using a common analytic framework, this study collected and analyzed policies and curricula on mental health literacy from the three countries (Philippines, 22; Indonesia, 9; and Japan, 6). The basic education curricula developed by their respective Ministries of Education were used.

**Results:**

This study highlights two key findings on school-based mental health literacy. First, mental health literacy is incorporated into health and physical education in Japan, health, values education and homeroom guidance in the Philippines, and religious education in Indonesia. Second, while the Philippines and Indonesia implement mental health education based on established policies, Japan lacks a core mental health literacy policy but has developed and implemented related curricula through its course of study guidelines. The curriculum analysis identified a specific challenge: a lack of “*first aid skills to support others who are developing a mental disorder or are in a mental health crisis*”.

**Conclusions:**

This study revealed the partial implementation of mental health literacy education in the Philippines, Indonesia, and Japan. The Philippines offers a nearly comprehensive curriculum on mental health literacy (grades 1–12), Japan incorporates it into health education (grades 5–10), and Indonesia integrates it into religious education (grades 1–12). While the Philippines and Indonesia align with mental health policies, Japan relies on its national curriculum without a core policy. A key challenge was indicated involving teachers, guidance counselors, or school health personnel as key actors to support students with mental disorders or those potentially at risk, as well as to handle emergency cases of mental disorders in schools. Recommendations include systematic monitoring of the implementation of school-based mental health policies, collaboration with UN agencies to align with international standards while incorporating culturally tailored strategies for each country.

## Introduction

Managing mental health services, support, and curriculum in educational settings has gained increasing importance in both public health and educational policy. In the Western Pacific Region, the burden of mental disorders such as anxiety, conduct disorders, and eating disorders among children and adolescents has increased in 2019 [1]. In Japan and China which are located in the Western Pacific Region, the suicide rate was highest among children and adolescents in 2020 when COVID-19 was starting to spread [2–4]. Furthermore, a previous study in the US found that given the high and rising rates of mental disorders in young children following the COVID-19 pandemic, enhancing the availability and accessibility of mental health services for those in need is essential [5].

Mental health literacy, as defined by Jorm (1997), refers to “*knowledge and beliefs about mental disorders that aid in their recognition, management, or prevention*” [6]. The significance of this field is particularly notable for children and adolescents, who are in their critical developmental stage. This stage also coincides with the age of onset of most mental disorders. Furthermore, Jorm distinguished several components of mental health literacy in 2012: (a) *knowledge of how to prevent mental disorders*, (b) *recognition of when a disorder is developing*, (c) *knowledge of help-seeking options and treatments available*, (d) *knowledge of effective self-help strategies for milder problems*, and (e) *first aid skills to support others who are developing a mental disorder or are in a mental health crisis* [7]. School-based mental health literacy aims to improve knowledge and beliefs about mental disorders among school-aged children through curricular interventions.

The World Health Organization (WHO) has been active in promoting mental health in schools as part of its broader global mental health strategy [8, 9]. The WHO has identified several components for the prevention of mental illness among school-aged children. These include the (1) promotion of mental health awareness; (2) integration of mental health into school curricula; (3) creation of supportive school environments; (4) provision of access to mental health services; and (5) enhancement of educators’ capacity. Global mental health strategy has not yet been adapted to the context of each country, which has not yet reflected variations in the educational system, culture and religion. The WHO stated the strategy for mental health to provide their services and this could be addressed in the country context. While these principles guide its approach to mental health promotion in schools, specific strategies and interventions may vary according to the cultural, social, and economic context of different regions and countries. The global policy review highlights the critical role of governments, international agencies, and donors in supporting schools to create learning and social environments that promote mental health and well-being. A key priority is for policymakers to establish country-level policies, operational guidelines, and regulations to address the full spectrum of mental health needs, including promotion, prevention, and access to services both in schools and the community. Consequently, the WHO provides guidelines and recommendations that can be adapted at the national, regional, and local levels to address the specific contexts [10]. Instead, the WHO integrates mental health promotion into broader frameworks for promoting well-being and addressing mental health challenges in various settings, including Health Promoting Schools (HPS) [11].

A community-based approach to mental health care can enhance access to mental health services and reduce the risk of mental disorders, including drug and alcohol use, among school-aged children. The primary goal of mental health literacy interventions is to educate students, educators, and community members about the causes and risk factors of mental disorders, the ability to recognize symptoms, and the importance of mental health awareness [12]. Moreover, encouraging adolescents to seek help must be accompanied by expanded availability of mental health support for all those in need; however, this remains a significant challenge for child and adolescent mental health services [13]. In terms of interventions, the WHO has promoted the integration of mental health care services into primary health care services, with the objective of improving access to mental health care in community-based settings [14]. Furthermore, the WHO emphasized that adolescents, who are at an increased risk of alcoholism or non-communicable diseases due to high rates of tobacco and alcohol consumption, require not only physical but also mental health care, as they have a high potential for developing mental disorders. A global policy review on school-based mental health promotion in 2023 revealed that one of the commonalities among the reviewed policies was the recommendation to implement school-based mental health in the community with the involvement of parents based on HPS [10]. This involves school community members such as students, teachers, and parents. However, this global policy review found that guidelines by the United Nations (UN) emphasize universal interventions that are aligned with the comprehensive school health approach, but lack focus on specific topics such as mental health [10]. One of the components of the HPS is creating a supportive environment.

The UN has made concerted efforts to create guidelines and recommendations for the prevention of mental illness among adolescents, and this has been updated to promote school mental health services. In 2020, the WHO published a guideline entitled “Guidelines on Mental Health Promotive and Preventive Interventions for Adolescents” to provide evidence and recommendations for the prevention of mental illness among adolescents [15]. Furthermore, in 2023, the WHO and United Nations Children’s Fund (UNICEF) launched the Helping Adolescents Thrive (HAT) initiative, with the objective of enhancing positive mental health, preventing self-harm, and reducing mental illness among adolescents [16]. In 2023, despite a decline in suicide rates in many countries during the COVID-19 pandemic, suicide remained one of the leading causes of death among adolescents worldwide. In response, the Global Accelerated Action for the Health of Adolescents (AA-HA!) initiative was updated, placing a particular focus on improving adolescent mental health [17]. Consequently, the WHO has published multiple guidelines regarding the common obstacles to mental health among adolescents. Given the WHO’s initiatives and policy documents, it is important to understand the interpretation and application of this support at national (and even more local) levels [14].

A previous study in Canada highlights that incorporating a mental health literacy resource into the curriculum, when delivered by regular classroom teachers, can significantly and sustainably enhance mental health knowledge and reduce stigmatizing attitudes among high school students [18]. Although mental health services including guidance documents were promoted globally, only a few studies were conducted to clarify the implementation of school-based mental health literacy in Asia. A study in Vietnam and Cambodia supported the value of school-based mental health literacy training implemented in the classroom through in-service teacher training programs [19]. A study in Manila, Philippines, assessed teachers’ depression and suicide literacy, revealing varied levels of knowledge about suicidal behaviors [20]. Most respondents preferred “informing the parents” as their primary referral method. The findings highlight the need for targeted mental health literacy interventions to enhance teachers’ competency in addressing depression and suicide. A study in Indonesia examined the mental health literacy of Indonesian children and young people and their parents, revealing low level of them. Emphasis on religiosity, spirituality, and self-responsibility led to self-blame, reliance on traditional healers, and reduced professional help-seeking [21]. Culturally tailored strategies are needed to reduce stigma, improve mental health literacy, and promote effective help-seeking, focusing on families and younger generations. A previous study on the evaluation of school mental health literacy education in Japan, highlighted that early intervention is essential to prevent mental illness among adolescents [22]. The study also indicated that the recently introduced course of study related to mental illness should be enhanced to contribute to the prevention of increased mental illness among adolescents. In Japan, “Course of Study” represents a national curriculum standard set by the Ministry of Education, Culture, Sports, Science, and Technology (MEXT). The Course of Study sets national standards, balancing foundational skills, moral education, and active learning, regularly updated to reflect societal needs. Each curriculum aligns with national priorities while preparing students for global challenges. However, the health and physical education curriculum for high school students in Japan is dependent on the newly introduced course of study. This indicates that it has yet to reflect in its curriculum the international approach as recommended in the WHO global mental health strategy.

This study aimed to compare the policies and curricula formulated in the Philippines, Indonesia, and Japan, and to make recommendations for the future dissemination of school-based mental health literacy in Asian countries through a comparative analysis of three Asian countries that have enacted mental health-related legislation as a national policy and established curricula on mental health literacy.

## Methods

This study used deductive content analysis to examine both policies and basic education curricula related to school-based mental health literacy in three Asian countries—the Philippines, Indonesia, and Japan—which have successfully enacted and implemented mental health laws or policies addressing the needs of both adults and school-aged children. These three countries, each with their own unique socio-cultural environment, including their approach to education, offer a rich tapestry of strategies and methodologies for addressing mental health literacy. Table 1 shows a comparison of the characteristics of the target countries. This study analyzed school-based mental health literacy policies and basic education curricula from the Philippines, Indonesia, and Japan using deductive content analysis. Policies were examined with the policy triangle framework and global mental health policies, while the basic education curricula were analyzed based on prior studies. The collected documents of this study analyzed both policies and basic education curricula that were adapted to the deductive content analysis methods. Policies were analyzed using the policy triangle framework [23] and mapped using the review points [10]. Basic education curricula were analyzed using the definition of mental health literacy [7].

**Table 1:** Characteristics of three Asian countries and government branches responsible for school-based mental health literacy

	Philippines	Indonesia	Japan
Population (World Bank, 2023)	117,337.37	277,534.12	124,516.65
Income classification (World Bank, 2023)	Lower-middle income	Upper-middle income	High income
Official language	Filipino	Bahasa Indonesia	Japanese
Education system	Centralized	Decentralized	Centralized
Responsible for mental health programs and services	Department of Education, Department of Health, Local Government Units	Ministry of Health, Ministry of Education and Culture, Ministry of Social and Welfare	Ministry of Health, Labour and Welfare
Multisector involvement	Philippine Council for Mental Health	Ministry of Education and Culture, Ministry of Religious, NGOs	Ministry of Education, Culture, Sports, Science and Technology (MEXT)

Population (World Bank, 2023): https://data.worldbank.org/indicator/SP.POP.TOTL

Income classification (World Bank, 2023): https://blogs.worldbank.org/en/opendata/new-world-bank-group-country-classifications-income-level-fy24

### Progress of the National Center for Global Health and Medicine (NCGM) project on mental health

The NCGM project, launched in 2017, initially focused on mental health training for educators, health workers, and physicians in Japan and the Philippines [24]. In 2020, it expanded to address mental health promotion challenges through collaboration with the University of the Philippines Manila [25]. By 2022, the “School Mental Health Project for Children and Adolescents Post-COVID-19 Pandemic” advance school mental health initiatives, particularly in developmental disorders. In 2023, the project became a tri-nation collaboration, including Indonesia. In 2024, the project convened a meeting to standardize school mental health literacy education across Southeast Asian countries.

### Data collection

The policy document review identified recommendations for policy options for school-based mental health literacy by conducting a comparative analysis among Asian countries with similar populations. These included the Philippines, Indonesia, and Japan. The curriculum review was conducted to describe the national curricula on mental health literacy education in the selected countries. This study acknowledged potential limitations, such as incomplete access to policy documents or curricula, which may have resulted in gaps in the analysis. To address these issues, efforts were made to obtain comprehensive data by consulting official government sources, education departments, and publicly available databases.

The collection and review of documents was conducted through three meetings in November 2023, February 2024, and June 2024. These meetings were organized by the Department of Child and Adolescent Psychiatry of Kohnodai Hospital, National Center for Global Health and Medicine in Japan. NCGM has contributed to the promotion of mental health among children and adolescents through the Program for International Promotion of Japan’s Healthcare Technologies and Services. An overview of the NCGM project is provided below. After collection, all data were analyzed to compare findings across the three Asian countries, including policies and curriculum.

### Policy document review

The policy document review was conducted to identify the legal and policy foundation that shapes school-based mental health literacy. Documents may be used as part of a systematic evaluation within a study, and they may take a variety of forms [26]. This study collected policies and curricula on mental health literacy based on the common framework among the three countries and analyzed the policies (the Philippines, 22; Indonesia, 9; and Japan, 6) and curricula that were constructed by the Ministry/Department of Education in each country. The policies were extracted from the collected documents using a stepwise process as follows:

1. Policy collection

The collection of policies was carried out according to the following keywords: (school health OR health promoting school) AND (mental health) AND (law* OR policy* OR regulation*). This was followed by Google searches with regional settings applied, using the same search terms along with the country name (e.g., ‘search terms’ and Japan). Official government websites and inputs from local mental health experts were also utilized to search for pertinent policies. The keywords were used to search for relevant policies in the local language such as in Japanese for Japan and Bahasa Indonesia for Indonesia. In the Philippines, the policies were searched in English. Data were extracted from school health/mental health policies released between 2000 and 2023.

(2) Translation and policy screening

The collected policies from Japan and Indonesia were translated into English from the local language using a translation function (DeepL Translator) to maintain the accuracy and quality of translation. After collecting the policies, the researchers used a translation function to interpret their contents, and this confirmed the accuracy among the researchers. Relevant policy documents on mental health were collected and classified into the framework and were extracted according to policy definitions stated by the Centers for Disease Control and Prevention (CDC) [27]. The collected policies were screened and the type of documents based on the policy definition by CDC including a law, regulation, procedure, administrative action, incentive, or voluntary practice of governments and other institutions throughout the policy screening.

(3) Summary of the collected policies

The titles of the collected policies related to school-based mental health literacy among three Asian countries are summarized in Table 2. Also, the details of collected policies in each country are summarized in Table 3 (Philippines), Table 4 (Indonesia), and Table 5 (Japan).

**Table 2:** Overview of the reviewed policies on school-based mental health literacy

	No.	Philippines	Indonesia	Japan
Policy documents	1	Administrative Order No. 8 s.2001 National Mental Health Policy (2001, Department of Health)	Regulation of the Minister of Education and Culture of The Republic of Indonesia No. 111/ 2014 on Guidance and Counseling in Primary Education and Secondary Education	Act on Mental Health and Welfare for the Mentally Disabled (1950, Ministry of Health, Labour and Welfare)
	2	Administrative Order No. 2016-0039 Revised Operational Framework for a Comprehensive National Mental Health Program (2016, Department of Health)	Minister of National Education Republic of Indonesia Regulation No. 57 Year 2009 Concerning Helping for the Development of Health School	School Health and Safety Act (1958, Ministry of Health, Labour and Welfare)
	3	Republic Act No. 11036 Mental Health Act (2018, Seventeenth Congress of the Philippines) and its Implementing Rules and Regulations	Minister of Health Republic of Indonesia’s Decision No HK.01.07/MENKES/2015/2023 Regarding Technical Instructions for the Integration of Primary Health Services	School Health and Safety Act Enforcement Regulation (1958, Ministry of Health, Labour and Welfare)
	4	Republic Act 11,223 Universal Health Care and its Implementing Rules and Regulations	Law document No.18/2014 About Mental Health	Basic Education Act (1947, Ministry of Education, Culture, Sports, Science and Technology)
	5	Guidelines on the Healthy Settings Framework in Learning Institutions	Regulation of Minister of Health No. 25/2014 about Child Health Efforts/Initiatives	Developmentally Disabled Persons Support Act (2004, Ministry of Health, Labour and Welfare)
	6	DepEd Memorandum No. 074 s. 2021 Inclusion and Promotion of Mental Health in all DepEd events and programs	Law Document No. 35/2014 about Child Protection	Act Establishing the Children and Families Agency (Act No. 75 of 2022, Children and Family Agency)
	7	Republic Act No. 9165 The Comprehensive Dangerous Drugs Act of 2022	Regulation of the Minister of Education and Culture No. 46/2023 About the Prevention and Management of Violence in Education Environment	
	8	Magna Carta for Disabled Persons	Decree of the Minister of Health Republic of Indonesia Number HK.02.02/MENKES/73/2015 Regarding to Mental Medicine Service National Guideline	
	9	Republic Act No. 11476 GMRC and Value Education Act (2020, Eighteenth Congress of the Philippines)	Republic of Indonesia’s Law No. 17 Year 2023 Concerning Health	
	10	Ok sa DepEd: School Mental Health Program		
	11	Child Protection: RA 7610		
	12	[Republic Act 8980] An Act Promulgating A Comprehensive Policy And A National System For Early Childhood Care And Development (Eccd), Providing Funds Therefor And For Other Purposes		
	13	DepEd Task Force COVID-19 Memorandum No. 82 (2020) Mental Health and Psychosocial Support Services for Learners, Parents, and DEPED Personnel, and Printing of MHPSS Materials		
	14	Republic Act No. 10354, or the Responsible Parenthood and Reproductive Health Act of 2012		
	15	DepEd Order 32 s. 2017: Gender Responsive Basic Education Policy		
	16	DepEd Order No. 044 s. 2021 Policy Guidelines on the Provision of Educational Programs and Services for Learners with Disabilities in the K to 12 Basic Education Program		
	17	Memorandum DM -OUCI-2021–055 Guidelines on the Counseling and Referral System of Learners for S.Y. 2020–2021		
	18	DM-OUCI-2021- 144—Implementation of Homeroom Guidance (HG) during Crisis Situation		
	19	DepEd Memorandum No. 071 s. 2021 Preparations for the Pilot Face-to-Face, Expansion and Transitioning to New Normal		
	20	DepEd DOH JMC No. 01 s. 2021 Operational Guidelines on the Implementation of Limited Face-to-Face Learning Modality		
	21	Joint Memorandum Circular No. 001 s. 2022 Revised Operational Guidelines on the Progressive Expansion of Face to Face Learning Modality		
	22	DO No. 23, s. 2022—Child Find Policy for Learners with Disabilities Towards Inclusive Education

**Table 3:** Philippines national policies mapped on school-based mental health literacy

Name	Issuing authority	Year	Type of document	Reference number/URL
Enhanced Basic Education Act (RA 10533)	Philippine National Government	2013	Republic Act	https://issuances-library.senate.gov.ph/sites/default/files/2022-10/20130515-RA-10533-BSA.pdf
An Act Institutionalizing Good Manners And Right Conduct And Values Education In The K To 12 Curriculum, Appropriating Funds Therefor, And For Other Purposes (RA 11476)	Philippine National Government	2020	Republic Act	https://lawphil.net/statutes/repacts/ra2020/ra_11476_2020.html
Oplan Kalusugan (OK) sa DepEd policy—DO No. 28 s. 2018	Department of Education	2018	Department Order	https://www.deped.gov.ph/wp-content/uploads/2018/07/DO_s2018_028.pdf
DepEd Child Protection Policy (DO No. 20 s.2012)	Department of Education	2012	Department Order	https://www.scribd.com/document/360567074/DepED-Child-Protection-Policy
An Act Providing For Stronger Deterrence And Special Protection Against Child Abuse, Exploitation And Discrimination, And For Other Purposes RA 7610	Philippine National Government	1992	Republic Act	https://lawphil.net/statutes/repacts/ra1992/ra_7610_1992.html
An Act Promulgating A Comprehensive Policy And A National System For Early Childhood Care And Development (Eccd), Providing Funds Therefor And For Other Purposes	Philippine National Government	2000	Republic Act	https://lawphil.net/statutes/repacts/ra2000/ra_8980_2000.html
DepEd Task Force COVID-19 Memorandum No. 82 (2020) Mental Health and Psychosocial Support Services for Learners, Parents, and DEPED Personnel, and Printing of MHPSS Materials	Department of Education	2020	Department Memorandum	https://www.deped.gov.ph/wp-content/uploads/2020/08/DTFC-Memo_82_Mental-Health-and-Psychosocial-Support-Services-for-Learners-Parents-and-DepEd-Personnel-and-Printing-of-MHPSS-Materials_2020804_Final_1597035517.pdf
Magna Carta for Disabled Persons	Philippine national government	1992	Republic Act	http://hrlibrary.umn.edu/research/Philippines/RA%207277%20-%20Magna%20Carta%20of%20Disabled%20Persons.pdf
An Act Establishing a National Mental Health Policy for the Purpose of Enhancing the Delivery of Integrated Mental Health Services, Promoting and Protecting the Rights of Persons Utilizing Psychosocial Health Services, Appropriating Funds Therefor and Other Purposes (RA No. 11036)	Philippine National Government	2017	Republic Act	https://lawphil.net/statutes/repacts/ra2018/ra_11036_2018.html

**Table 4:** Indonesia national policies mapped on school-based mental health literacy

Name	Issuing authority	Year	Type of document	Reference number/URL
Mental Health	President of the Republic of Indonesia and Council of People's Representatives (DPR) of the Republic of Indonesia	2014	Law	https://peraturan.bpk.go.id/Details/38646/uu-no-18-tahun-2014
Health	President of the Republic of Indonesia and Council of People's Representatives (DPR) of the Republic of Indonesia	2023	Law	https://peraturan.bpk.go.id/Details/258028/uu-no-17-tahun-2023
Child Protection	President of the Republic of Indonesia and Council of People's Representatives (DPR) of the Republic of Indonesia	2014	Law	https://peraturan.bpk.go.id/Details/38723/uu-no-35-tahun-2014
Child Health Efforts Initiatives	Minister of Health	2014	Ministry regulation	https://peraturan.bpk.go.id/Home/Download/108349/Permenkes%20Nomor%2025%20Tahun%202014.pdf
The Prevention and Management of Violence in Education Environment	The Minister of Education and Culture	2023	Ministry regulation	https://jdih.kemdikbud.go.id/sjdih/siperpu/dokumen/salinan/salinan_20230818_101558_2023pmkemdikbud46.pdf
Concerning to Providing Assistance for the Development of Health School	Minister of National Education Republic of Indonesia	2009	Ministry regulation	https://simpuh.kemenag.go.id/regulasi/permendiknas_57_09.pdf
Regulation Of the Guidance and Counseling	Ministry of Education and Culture Of The Republic Of Indonesia	2014	Ministry regulation	https://jdih.kemdikbud.go.id/sjdih/siperpu/dokumen/salinan/Permendikbud%20Nomor%20111%20Tahun%202014.pdf
Guidance and Development of School Health Unit (UKS)	Ministry of education and culture, minister of health, Minister of religion, and Minister of the republic of Indonesia	2014	Joint regulation	https://simpuh.kemenag.go.id/regulasi/pb4menteri_2014.pdf
Mental Medicine Service National Guideline	The Minister of Health	2015	Decree of minister	http://hukor.kemkes.go.id/uploads/produk_hukum/KMK_No._HK_.02_.02-MENKES-73-2015_ttg_Pedoman_Nasional_Pelayanan_Kedokteran_Jiwa_.pdf
Protection and Compliance Rights of Persons with Disabilities	Province of the Special Region of Yogyakarta	2012	Regional regulations	https://peraturan.bpk.go.id/Home/Details/25643
Technical Instructions for the Integration of Primary Health Services	Minister of Health	2023	Technical instructions	https://kesmas.kemkes.go.id/konten/159/0/petunjuk-teknis-integrasi-pelayanan-kesehatan-primer
Guideline for the Implementation of Mental Health in First Level Health Facilities	Ministry of Health	2020	Manual guideline	https://ayosehat.kemkes.go.id/buku-pedoman-penyelenggaraan-kesehatan-jiwa-di-fasilitas-kesehatan-tingkat-pertama
Guideline for the Implementation of Mental Health in First Level Health Facilities	Ministry of Health	2020	Manual guideline	
SIMKESWA (Mental Health Information System)	Ministry of Health	2022	Manual book	https://simkeswa.kemkes.go.id/assets/BUKU%20PETUNJUK%20PENGGUNA%20SIMKESWA%202022%201.1-REV.pdf
Guideline for Mental Health and Psychosocial Support during the COVID-19 Pandemic	Ministry of Health	2020	Manual guideline	https://infeksiemerging.kemkes.go.id/document/download/vBr
Technical Instruction for Implementing the National Nutritional Action Movement	Ministry of Health	2022	Technical instruction	https://ayosehat.kemkes.go.id/buku-petunjuk-teknik-gerakan-nasional-aksi-bergizi
Stop Bullying Modul	Ministry of Education, culture, research, and technology	2021	Modul	https://ditpsd.kemdikbud.go.id/upload/filemanager/download/pencegahan-3-dosa-besar-pendidikan/20210308%20Buku%20Saku-Stop%20Bullying.pdf

**Table 5:** Japan national policies mapped on school-based mental health literacy

Name	Issuing authority	Year	Type of document	Reference number/URL
Act on Mental Health and Welfare for the Mentally Disabled (1950, Ministry of Health, Labour and Welfare)	Ministry of Health, Labour and Welfare	1950	Act	https://laws.e-gov.go.jp/law/325AC0100000123/20230401_504AC0000000104
School Health and Safety Act (1958, Ministry of Health, Labour and Welfare)	Ministry of Health, Labour and Welfare	1938	Act	https://laws.e-gov.go.jp/law/333AC0000000056
School Health and Safety Act Enforcement Regulation (1958, Ministry of Health, Labour and Welfare)	Ministry of Health, Labour and Welfare	1958	Act	https://www.mhlw.go.jp/stf/shingi/2r9852000002mcip-att/2r9852000002mdgz.pdf
Basic Education Act (1947, Ministry of Education, Culture, Sports, Science and Technology)	Ministry of Education, Culture, Sports, Science and Technology (MEXT)	1947	Act	https://www.mext.go.jp/b_menu/hakusho/html/others/detail/1317990.htm
Developmentally Disabled Persons Support Act (2004, Ministry of Health, Labour and Welfare)	Ministry of Health, Labour and Welfare	2004	Act	https://www.mext.go.jp/a_menu/shotou/tokubetu/main/1376867.htm
Act Establishing the Children and Families Agency (Act No. 75 of 2022)	Children and Family Agency	2022	Act	https://www.cfa.go.jp/en/act-establishing-the-CFA-en

### Curriculum review

School curricula related to mental health literacy were extracted in each country and were reviewed which targeted school-aged children who are from elementary schools to high schools. The Philippines, Indonesia, and Japan employ distinct national curricula to address educational goals. The Philippines has implemented the K-12 curriculum, a comprehensive framework covering Kindergarten to Grade 12 emphasizing core competencies, career readiness, and specialized senior high school tracks for holistic development. In Indonesia, the curriculum fosters thematic learning, incorporating science, technology, engineering, arts, and mathematics (STEAM). The “2013 School Curriculum (Kurikulum 2013 Sekolah)” focuses on competency-based learning, integrating character education, STEAM, and critical thinking, with accessible guidelines for uniform implementation. The total number of curricula was indicated in the curriculum review of the Methods section as follows: Philippines: Elementary School (20), Junior High School (12), Senior High School (4) (Table 9); Indonesia: Primary schools (3), Middle Schools (2), Senior High Schools (3) (Table 10); Japan: Elementary School (5), Junior High School (7), High School (9) (Table 11).

1. Curriculum collection

The K-12 curricula in Japan and Indonesia which are written in their respective local dialects and the curriculum of the Philippines, which was in English, were collected for this study.

(2) Curriculum screening

The reviewed curriculum was extracted by referring to the guidelines for K-12 curricula in the Philippines, Indonesia, and Japan.

### Data analysis

The data collected were analyzed separately for both the policies and the curricula using a deductive content analysis approach [28, 29]. The policies in the three Asian countries were analyzed using two matrices. The first matrix (Table 6) examined policy content and actors using the policy triangle framework [23]. The extracted policies were examined through two policy-making elements, specifically the actors and content (Table 6), according to the policy triangle framework of (i) processes, (ii) actors, (iii) content, and (iv) context [23].

**Table 6:** Overview of the policy content analysis for school-based mental health literacy education

Country	Actors		Contents
Philippines	1	Department of Health (DOH)	*Community or Recovery-based approach to treatment: The *Department of Health (DOH)* shall support program integration across different levels of health care from community based to facility-based services, as well as different settings (home, schools, workplace, industry) that facilitate the continuum and complementation of care.* (Revised Operational Framework for a Comprehensive National Mental Health Program) *The *Department of Health (DOH)* shall develop alternatives to institutionalization, particularly community, recovery-based approaches to treatment aimed at receiving patients discharged from hospitals, meeting the needs expressed by persons with mental health conditions, and respecting their autonomy, decisions, dignity, and privacy.* (Mental Health Act) *The*Department of Health (DOH)* shall improve research capacity and academic collaboration on national priorities for research in mental health, particularly operational research with direct relevance to service development, implementation, and the exercise of human rights by persons with mental health conditions, including establishment of centers of excellence.* (Mental Health Act)
	2	Department of Social Welfare and Development (DSWD)	*The *Department of Social Welfare and Development (DSWD)*, in coordination with the *Local Government Units (LGUs)* and the *DOH*, formulate, develop, and implement community resilience and psychosocial well-being training, including psychosocial support services during and after natural disaster and other calamities.* (Mental Health Act)
	3	Local Government Units (LGUs)	
	4	Teacher	*Counseling and Referral: Counseling and referral services shall be made available for all learners…. *The teacher* shall accomplish a referral form that would be submitted to the Guidance Counselor. Upon receipt of the referral form, *the Guidance Counselor* shall assess, evaluate, and provide the schedule for intake interview or other services that may be deemed necessary to address the needs of the learners.* (Guidelines on the Counseling and Referral System of Learners for S.Y. 2020–2021)
	5	Guidance Counselor	
	6	Mental health facilities	*In close coordination with *mental health facilities*, *academic institutions*, and other stakeholders,*mental health professionals*, workers, and other service providers shall undergo capacity building, reorientation, and training to develop their ability to deliver evidence-based, gender-sensitive, culturally appropriate and human rights-oriented mental health services, with emphasis on the community and public health aspects of mental health*. (Mental Health Act)
	7	Academic institutions	
	8	Mental health professionals	
	9	Department of Education (DepEd)	*Dissemination and Enlightenment to the Public: This memorandum also includes] a poster containing helplines from different organizations to provide learners and *Department of Education (DepEd)* personnel ways to seek help and persons to contact in times of mental and psychological distress.* (Inclusion and Promotion of Mental Health in all DepEd events and programs)
	10	Commission on Higher Education (CHED)	Evidence-based mental health programs: *The *Department of Education (DepEd), Commission on Higher Education (CHED), and Technical Education and Skills Development Authority (TESDA)* shall develop guidelines and standards on age-appropriate and evidence-based mental health programs both in public and private institutions.* (Mental Health Act) *Integration of Mental Health into the educational system: The *Department of Education (DepEd), CHED, and TESDA* shall Integrate age-appropriate content pertaining to mental health into curriculum at all educational levels both in public and private institutions.* (Mental Health Act)
	11	Technical Education and Skills Development Authority (TESDA)	
	12	Educational institutions	*Mental Health Promotion in Educational Institutions: *Educational institutions* shall develop policies and programs for students, educators, and other employees designed to raise awareness on mental health issues, identify and provide support and services for individuals at risk, and facility access, including referral mechanisms of individuals with mental health conditions to treatment and psychosocial support. (Mental Health Act)*
Indonesia	1	Educational institution	*Mental Health awareness and Promotion in Educational Institutions: *Educational institutions* (under *the Ministry of Education and Culture and the Ministry of Religious Affairs*) have developed policies focusing on self-awareness of mental health problems and their management and also facilitating mental health promotion among students.* (Regulation Of the Minister of Education and Culture of the Republic of Indonesia No. 111/ 2014) *Mental health environment and infrastructure: *Educational Institutions* (under *the Ministry of Education and Culture and the Ministry of Religious Affairs*) have developed the regulation on creating a healthy school environment within the broader educational frameworks to support student’s mental health and well-being. This regulation also explains healthy school guidance*. (Minister of National Education Republic of Indonesia Regulation No. 57/2009)
	2	Ministry of Health	*Capacity building and community engagement: The *Ministry of Health* on Decision No.HK.01.07/MENKES/2015/2023 has a program on training providers in mental health care and engaging the community in mental health promotion efforts. The Law no. 18/2014 About Mental Health highlights the capacity building program for health care professionals to address children's mental health needs and also community involvement as a support system*
	3	Primary Health Care Center	*Integrating primary health services and children’s mental health: The *Ministry of Health* as a regulator and *Primary Health Care Center* as a program executor including comprehensive services on mental health, preventive and promotive services, curative and rehabilitative care. A cross-sector coordination also arose involving healthcare providers, *educational institutions*, and community, including *religious figures* and *community leaders*.* (Minister of Health Republic of Indonesia’s Decision No. HK.01.07/MENKES/2015/2023)
	4	Religious figures	
	5	Community leaders	
	6	National and local government	*Dissemination and enlightenment of knowledge in mental health care services* The national and local government* shall develop a system focusing on mental health promotion and prevention as well as curative and rehabilitative programs, including access to care services.* (Minister of Health Republic of Indonesia’s Decision No. HK.01.07/MENKES/2015/2023; Law document No.18/2014; Regulation of Minister of Health No. 25/2014)
Japan	1	Citizen	Citizens* shall endeavor to maintain and promote mental health, deepen their understanding of persons with mental disorders…* (Act on Mental Health and Welfare for the Mentally Disabled. 1950. Ministry of Health, Labour and Welfare)
	2	Yogo Teacher (School Nurse)	*Health Guidance: Article 9* YogoTeacher (School nurse)* and other personnel shall, in cooperation with each other, ascertain the mental and physical conditions of pupils,* etc.,* by means of health consultation or daily observation of their health conditions, and when they find that there is a health problem, provide necessary guidance to said pupils, *etc*.* (School Health and Safety Act. 1958. Ministry of Health, Labour and Welfare)
	3	Medical institutions	*Cooperation with Local Medical Institutions, *etc*.: Article 10 When providing first aid, health counseling, or health guidance, schools shall endeavor to cooperate with *medical institutions* and other relevant organizations in the area where said schools are located, as necessary.* (School Health and Safety Act. 1958. Ministry of Health, Labour and Welfare)
	4	National and local governments	*Dissemination and Enlightenment to the Public: Article 21 *The national and local governments* shall conduct necessary publicity and other enlightenment activities through schools, communities, homes, workplaces and various other venues in order to deepen public understanding of the characteristics of individual developmental disabilities and other developmental disabilities.* (Developmentally Disabled Persons Support Act. 2004. Ministry of Health, Labour and Welfare) *Dissemination and Enlightenment of Knowledge to Persons Engaged in Medical or Health Care Services: Article 22 *The national and local governments* shall endeavor to disseminate and enlighten the knowledge necessary for the detection of developmental disabilities to those engaged in medical or health care services.* (Developmentally Disabled Persons Support Act. 2004. Ministry of Health, Labour and Welfare)
	5	School counselors	*Section 4: Staff, Article 65–3: *School counselors* shall engage in providing psychological support for children in elementary schools.* (School Education Act Enforcement Regulation. 2024 Revision, Ministry of Education, Culture, Sports, Science and Technology)
	6	School social workers	*Article 65–4: *School social workers* shall engage in support for the welfare of children in elementary schools.* (School Education Act Enforcement Regulation. 2024 Revision, Ministry of Education, Culture, Sports, Science and Technology)
	7	Children and Family Agency	*This Act establishes the *Children and Families Agency*, positioning it as an external organ of the Cabinet Office; the Children and Families* *Agency is charged with handling functions related to increasing the welfare and improving the health of children (meaning people in the process of physical and mental development; the same applies hereinafter) and their families, providing other such support for children's healthy growth and for parenting, and advocating for children, based on the importance of the family's role in child-rearing and on the principle of making considerations that give due weight to the views of the child in accordance with the child’s age and maturity, and that prioritize children's best interests, in order to bring about a society in which children can all grow up healthy as independent individuals; the Agency is also charged with assisting in the Cabinet functions associated with the Cabinet's specific essential policies that are relevant to the abovementioned duties, and the Act provides for the particulars of the functions under the Agency's jurisdiction and for the particulars of its organization.* (*Act Establishing the Children and Families Agency, Act No. 75 of 2022, Children and Family Agency)*

A content analysis was carried out based on the “policy triangle framework” developed by [23]

The second matrix (Table 7) assessed the policy context in terms of the “Summary of key points of global mental health guidelines and manuals of UN agencies” [10] in comparison with global recommendations. The curriculum analysis was performed deductively following the definition of mental health literacy by Jorm in 2012 [7] and summarized in Table 8. With regard to the consultation between the researchers and stakeholders, contributed to extracting the findings and identifying the policy contents such as differences or commonalities, and brushing up the process of the reviews during the meetings throughout the project.

**Table 7:** Evaluation of the reviewed national policies using key points of global UN agencies’ mental health guidelines and manuals

Summary of key points of global UN agencies' mental health guidelines and manuals (adapted from Margaretta et al. 2023)						Philippines	Indonesia	Japan
1. Who is involved?	1. The policies, manuals and guidelines were mainly developed by the government, without support from the international agencies (e.g., WHO, UNICEF), mental health experts, research centers (e.g., CDC, EDC), and other international agencies (e.g., World Bank, USAID)					P	Y	Y
2. Purpose	2. The primary purpose of the policies was to improve member states' regional and national capacities for promoting health in schools and the community Several manuals/policies have specific purposes, for addressing current or existing mental health challenges (e.g., bullying, suicide)					Y	Y	Y
3. Scope of mental health issues on mental disorders addressed	3. School mental health issues were conceptualized and addressed using a comprehensive health network, where mental health is considered part of general health, and interventions are orchestrated for obtaining health and educational outcomes					Y	Y	P
	School-based mental health promotion efforts were with the intention:	3.1. Improving skills-based health education				Y	Y	Y
		3.2. Creating a supportive environment for obtaining general health, mental health, and socio-emotional well-being				Y	Y	Y
		3.3. Targeting the management of specific mental health problems				Y	Y	Y
4. Targeted audiences	4. Most guidelines use a top-down approach for equipping stakeholders to make plans, facilitating the organization of policies and resources, and creating partnerships and strategies (monitoring-evaluation) for implementation at the school level Manuals often used a pragmatic approach for assisting school-level implementation and supporting dissemination strategies to wider school audiences					Y	P	Y
	Two sectors were targeted:		4.1. People who work with children and adolescents, including school communities, health providers, social organizations, families, and youth			Y	Y	Y
			4.2. Governments, policy, and decision-makers, including national-level and ministerial-level officials, non-governmental agencies, and community leaders			Y	Y	Y
5. Strategies	5. Generally, guidelines and manuals charted principals (e.g., policies, laws, guidelines), components, and schemes for schools to become an enabling context for comprehensive school health, such as promulgated by Health-Promoting Schools					P	Y	Y
	For implementing mental health and well-being strategies, two approaches were used:			5.1. Environment-centered, which aims to improve the educational atmosphere, provide students with opportunities to connect with healthy role models and school activities (e.g., consultation, teacher training), curriculum (e.g., physical and health education), and increase access to health care and services		P	P	Y
				5.2. Child-centered, which aims to manage existing mental health problems through universal/targeted/indicated interventions		P	N	N
6. Assessment	Measurement approaches could be classified into three levels:			6.1. Basic assessment, which focuses on measuring processes and outcomes		P	P	P
				6.2. Specified indicator assessment, which aims to assess inputs, processes, outcomes, outputs, and impacts		P	N	P
				6.3. Detailed and complex assessment, which uses an elaborated assessment strategy and often accompanies with tools for monitoring and evaluation		N	N	P
7. Dissemination	7. Most guidelines could be used as references to develop national-level policies and systems for school-based mental health promotion Manuals were generally designed to be further used for training (e.g., teacher training both pre-service and in-service) and dissemination (e.g., providing educational material to parents in the community), with some including booklets to enable wider dissemination					Y	Y	Y
8. Scope of intervention	8. The multi-tiered approach was the most referred framework to indicate the scope of mental health and well-being promotion for school-age children and adolescents From the universal approach (promotive), the targeted approach (preventive), and the indicated approach (response/treatment) Within this, there was an emphasis on encouraging promotive and preventive approaches, particularly around building health behaviors/skills, and creating healthy school environments					P	Y	Y
9. What schools can do	9. School can focus on building their capacity to be an enabling context for school mental health					Y	P	P
	Three broad activities can be undertaken:				9.1. Form an implementation team with multiple stakeholders	Y	P	P
					9.2. Map resources and current system—profile gaps between needs with current resources	Y	N	P
					9.3. Engage in partnership with policymakers, community, and related stakeholders	Y	P	P

*Y* yes, *P* partial, *N* no

**Table 8:** Overview of the K-12 curriculum on school-based mental health literacy

		Subject: content (Grade)		
Educational period	Definition of mental health literacy (Jorm. 2012)	Philippines	Indonesia	Japan
Elementary schools	(a) Knowledge of how to prevent mental disorders	Health Education: Positive Expression of Feelings (2) Health Education: Mental, emotional, and social health, Healthy and Unhealthy relationships, Mental, emotional, and social health concerns Preventing and Managing Mental, Emotional, and Social Health Concerns (5) Health Education: Changes during Puberty (including emotional and social changes) (5) Health Education: Nature and Effects of Gateway Drugs, Impact of the Use and Abuse of Gateway Drugs, Prevention and Control of Use and Abuse of Gateway Drugs (5)	Religious Education: Implementation of religious beliefs in managing emotions (3,5)	Health and Physical Education: Disease prevention; Smoking, drinking alcohol, drug abuse and health (6)
	(b) Recognition of when a disorder is developing	Values Education: Knowing Oneself (Strengths, Weaknesses, Feelings) (1,2) Health Education: Medicine misuse and abuse, Potential dangers associated with medicine misuse and abuse (4)	Religious Education: Self-awareness: self-observation, self-understanding, self-uniqueness (1,2,3,4,5)	Health and Physical Education: Mental health; Development of the mind, Close relationship between mind and body, Coping with anxiety and worries (5)
	(c) Knowledge of help-seeking options and treatments available	Health Education: Healthy School and Community Environments (including psychosocial environment) (6)		
	(d) Knowledge of effective self-help strategies for milder problems	Values Education: Self-Esteem (1,2,3) Values Education: Confidence, Fortitude (3) Values Education: Empathy, Faith, Hope, Charity, Spirituality (3,4,5) Values Education: Concern for others, Compassion, Charity, Respect (5) Values Education: Fortitude, Perseverance, Patience, Critical thinking, Open-mindedness, Patience, Calmness (4,5,6)	Religious Education: Management of conflict: Implementation of religious beliefs by developing good behaviors in face of a conflict as a preventive measure for bullying (2,3)	
	(e) First aid skills to support others who are developing a mental disorder or are in a mental health crisis			
Junior high schools	(a) Knowledge of how to prevent mental disorders	Health Education: Introduction of Mental Health (7) Health Education: Holistic Health (includes physical, mental/ intellectual, emotional, social, and moral-spiritual) (7) Health Education: Nutrition Problems of adolescents (includes eating disorders) (7) Health Education: Types and Management of Common Mental Disorders, identifying triggers and warning signs, prevention, coping, and treatment (7) Health Education: Gateway Drugs, Protective and Risk Factors in the Use of Cigarettes and Alcohol, Prevention and Control of Gateway Drugs (8)	Physical Education: Understanding the concept of association between degree of physical fitness and overall health (including mental health) (7)	Health and Physical Education: Origin of health and causes of disease/lifestyle and health: Rest, sleep, and health (7)
	(b) Recognition of when a disorder is developing	Health Education: Understanding stress, Common areas of stressors that affects adolescents (peer, family, school, community), coping with stress (7) Health Education: Types and Management of Common Mental Disorders, identifying triggers and warning signs, prevention, coping, and treatment (7) Values Education: Emotion, Violence in School (8)		Health and Physical Education: Coping with cravings and stress and mental health: Mental and physical relationships, Coping with cravings, Coping with stress (7)
	(c) Knowledge of help-seeking options and treatments available	Health Education: Coping with dying and death (7) Health Education: Types and Management of Common Mental Disorders, identifying triggers and warning signs, prevention, coping, and treatment (7)	Social Studies, Religious Education: Having sense of responsibility, care, confidence; Developing a healthy lifestyle; Understanding the responsibility of being a part of a community (7,8,9)	
	(d) Knowledge of effective self-help strategies for milder problems	Health Education: Development of self-awareness and coping skills (7) Health Education: Types of intentional injuries (includes bullying, suicide, domestic violence, sexual abuse, etc.), Prevention and management of intentional injuries (includes preventing self-harm, seeking help from trusted individuals and health professionals, etc.) (9) Health Education: Drug Scenario in the Philippines, Factors that influence substance use and abuse, Drugs/ Substances of abuse, harmful effects of drugs on the body (9)		
	(e) First aid skills to support others who are developing a mental disorder or are in a mental health crisis			
High schools	(a) Knowledge of how to prevent mental disorders	Health Education: Existing National Laws Related to Health Trends, Issues, and Concerns (including Comprehensive Dangerous Drugs Act of 2002) (10) Values Education: A Stand on Protecting oneself Against Sexual Abuse Towards a Healthy Self-Esteem and Promotion of Human Dignity (10) Personal Development: Mental Health and Well-being in Middle and Late adolescence (11,12)	Religious Education: Understanding characteristic of mature adults including the emotional aspects (10)	Health and Physical Education: Prevention and recovery from mental illness; Characteristics of mental illness, Coping with mental illness (10)
	(b) Recognition of when a disorder is developing	Values Education: Respect for Life (10) Personal Development: Emotional Intelligence (11,12)	Religious Education: Understanding suicide (11)	Health and Physical Education: Smoking, drinking alcohol, drug abuse and health: Smoking, drinking alcohol and health, Drug abuse and health (10)
	(c) Knowledge of help-seeking options and treatments available			
	(d) Knowledge of effective self-help strategies for milder problems	Personal Development: Coping with Stress in Middle and Late Adolescence (11,12) Personal Development: Personal Relationships (11,12)	Religious Education: Understanding character and self-control (10,12)	
	(e) First aid skills to support others who are developing a mental disorder or are in a mental health crisis			

The curriculum analysis was carried out using the framework of the definition of mental health literacy by Jorm in 2012 [4]

Philippines; K to 12 Basic Education Curriculum (Department of Education)

Indonesia; 2013 School Curriculum (Kurikulum 2013 Sekolah) (Ministry of Education and Culture)

Japan; Course of Study, Guideline of Health and Physical Education (Ministry of Education, Culture, Sports, Science and Technology)

### Policy content analysis

**Fig. 1:**
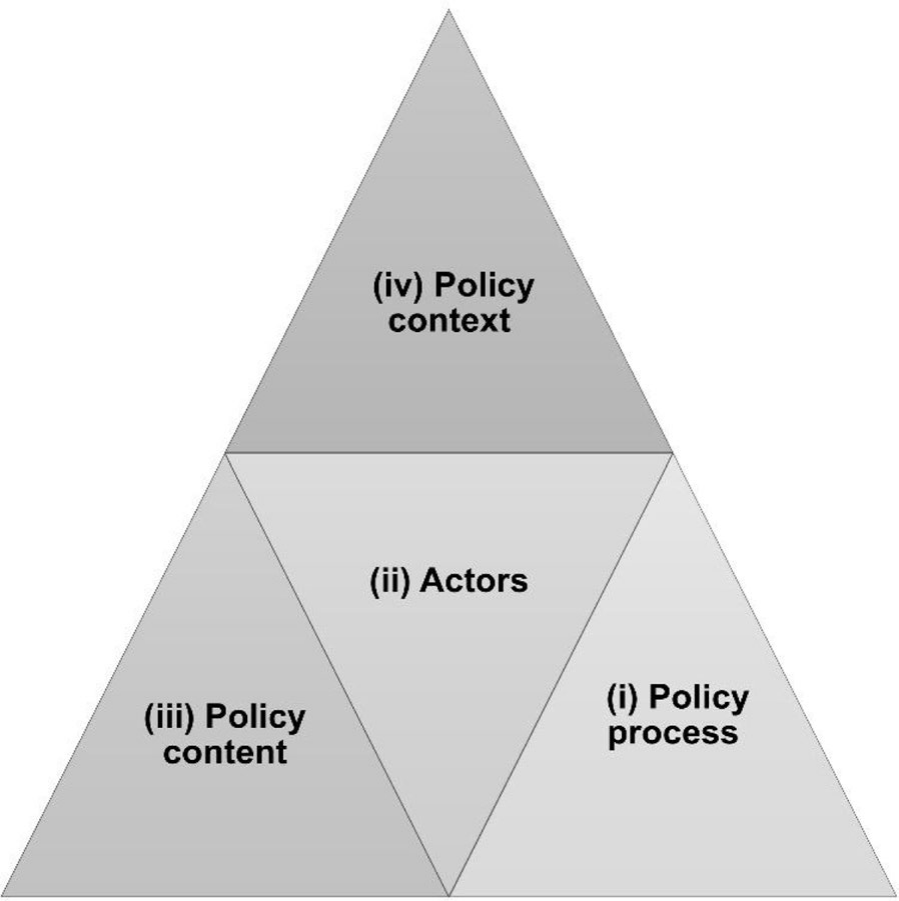
A model for health policy analysis [23]

Walt and Gilson stated in 1994 that understanding the process of health policy reform enables policymakers and researchers to plan more effectively for its implementation (Fig. 1). This framework allows for an in-depth understanding of the factors in the policy triangle by illustrating how they are interrelated. The triangle framework illustrates four dimensions and the linkages between them that should be considered when conducting policy analysis. In this study, two dimensions such as “content” and “actors” were applied to examine policy content and who implements it in each country. The content pertains to the substance of the policy proposal and the actors who are fundamental in formulating the policy and during policy implementation. The researchers reviewed the policies in each country (Tables 3, 4, 5) and clarified the “actors” and “contents” that are related to school-based mental health literacy (shown in Table 6) [30, 31].

### Policy mapping analysis

Table 7 was developed to assess the context of key national policies related to school-based mental health. The policies were assessed according to an adapted version of the “Summary of key points of global UN agencies’ “mental health guidelines and manuals” by Margaretta et al. in 2023 [10], which was compared with the global recommendations. The global policy reviews identified several commonalities among UN global policy documents on school mental health, such as the promotion of comprehensive school health and collaboration between schools, government, families, and communities, and this was summarized a total of 21 items that were used to assess the comprehensiveness of national policies relevant to school-based mental health (Table 7). The reviewers (co-authors) in each country consulted with their colleagues from government agencies or other experts and responded with changes through a consultative process [30, 31]. Information was abstracted from the identified policies in each country in Table 3 (Philippines), Table 4 (Indonesia), and Table 5 (Japan), relating to each of the 21 items. Policies were ranked (“Yes”, “Partial”, and “No”) based on how comprehensively they addressed each of the items in school-based mental health policies and manuals.

### Curriculum content analysis

The extracted curricula were analyzed deductively by referring to the definition of mental health literacy stated by Jorm in 2012 [7] and summarized in Table 8. Because the definition of mental health literacy curricula was not decided officially, thus, this study referred to the previous study in Japan by Ojio et al. in 2019 [22] to adapt the other countries such as the Philippines and Indonesia. This previous study indicated that the contents of the course of study in Japan were related to the concept of mental health literacy stated by Jorm et al., 1997 [1] and Kutcher et al., 2016 [32] following: (1) *mechanisms of mental illness, prevalence, onset age, risk factors, and treatability*, (2) *typical symptoms of mental health problems and illnesses*, (3) *self-help strategies for prevention of and recovery from mental illness*, (4) *enhancing help-seeking and helping behavior* and (5) *decreasing mental health-related stigma*. Deductive content analysis is applied when the structure of the analysis is operationalized based on prior knowledge [28, 29]. Accordingly, the curriculum was classified in consideration of the definition (concept) of mental health literacy stated by Jorm in 2012 [7] that were synthesized in Table 8, and it was reflected originality of this study to identify several challenges of this definition. Curriculum content analysis information was abstracted from the identified curricula in each country in Table 9 (Philippines), Table 10 (Indonesia), and Table 11 (Japan).

**Table 9:** Philippines national curriculum mapped on school-based mental health literacy

School category (total number of curricula)	Grade	Contents	Subject	Reference number/URL
Elementary school (20)	Grade 1	∙ Knowing Oneself (*Pagkilala sa sarili*) Strengths / potential (*kalakasan/ potensyal*) Weaknesses (*kahinaan*) Feelings (*damdamin*)	Edukasyon sa Pagpapakatao (Values Education)	https://www.bing.com/ck/a?!&&p=4f89c6484faba6c3b58746872b1263d5b53ace86d7da777eb00ff5a1cfee8977JmltdHM9MTczMzI3MDQwMA&ptn=3&ver=2&hsh=4&fclid=230f74d2-88b2-6a23-066d-662789586b9a&psq=ESP-CG.pdf+(deped.gov.ph)&u=a1aHR0cHM6Ly93d3cuZGVwZWQuZ292LnBoL3dwLWNvbnRlbnQvdXBsb2Fkcy8yMDE5LzAxL0VTUC1DRy5wZGY&ntb=1
	Grade 1	∙ Self-esteem (*Pagpapahalaga sa Sarili*) Pagtitiwala sa sarili (self-confidence)	Edukasyon sa Pagpapakatao (Values Education)	
	Grade 2	∙ Knowing Oneself (*Pagkilala sa sarili*) Strengths / potential (*kalakasan/ potensyal*) Weaknesses (*kahinaan*) Feelings (*damdamin*)	Edukasyon sa Pagpapakatao (Values Education)	
	Grade 2	∙ Self-esteem (*Pagpapahalaga sa Sarili*) Pagtitiwala sa sarili (self-confidence)	Edukasyon sa Pagpapakatao (Values Education)	
	Grade 2	∙ Positive Expressions of Feelings benefits of health expression of feelings expresses positive feelings in appropriate ways demonstrates positive ways of expressing negative feelings displays respect for the feelings of others	Health	https://www.deped.gov.ph/wp-content/uploads/2019/01/Health-CG_with-tagged-math-equipment.pdf
	Grade 3	∙ Self-Esteem (*Pagpapahalaga sa Sarili*)	Edukasyon sa Pagpapakatao (Values Education)	
	Grade 3	∙ Confidence (*Pagtitiwala sa Sarili*)	Edukasyon sa Pagpapakatao (Values Education)	
	Grade 3, 4, 5, 6	∙ Fortitude (*Katatagan ng loob*)	Edukasyon sa Pagpapakatao (Values Education)	
	Grade 3, 4	∙ Empathy (*Pagdama at pag-unawa sa damdamin ng iba*)	Edukasyon sa Pagpapakatao (Values Education)	
	Grade 3, 4, 5	∙ Pananalig sa Diyos (Faith) ∙ Pag-asa (Hope) ∙ Pagkakawanggawa (Charity) ∙ Ispiritwalidad (Spirituality)	Edukasyon sa Pagpapakatao (Values Education)	
	Grade 4	∙ Medicine Misuse and Abuse Self-Medication Improper use Dependence Addiction ∙ Potential Dangers Associated with Medicine Misuse and Abuse Physical harm such as deafness due to antibiotic misuse Psychological harm	Health	
	Grade 4,5,6	∙ Pagkamatiyaga (Perseverance) ∙ Pagkamapagtiis (Patience) ∙ Mapanuring pag-iisip (Critical thinking) ∙ Pagkakaroon ng bukas na isipan (Open-mindedness) ∙ Mapagpasensiya (Patience/Self-Control) ∙ Pagkamahinahon (Calmness)	Edukasyon sa Pagpapakatao (Values Education)	
	Grade 5	∙ Mental, Emotional and Social Health Characteristics of a Healthy Person (mentally, emotionally and socially) Ways to Develop and Nurture One’s Mental Health Ways to Stay Emotionally Healthy ∙ Healthy and Unhealthy relationships Signs of Healthy Relationships (loving family, genuine friendships) Importance of Healthy Relationships in Maintaining Health Signs of Unhealthy Relationships Managing Unhealthy Relationships ∙ Mental, Emotional and Social Health Concerns (include ways on how these negatively impact one’s health and well-being) Social anxiety Mood swings Teasing Bullying, including cyber bullying Harassment Emotional and physical abuse Other stressful- situations ∙ Preventing and Managing Mental, Emotional and Social Health Concerns Practicing life skills (communication/ assertiveness/ self-management/ decision-making) Finding Resources and Seeking help	Health	
	Grade 5	∙ Changes during Puberty includes Emotional and Social Changes	Health	
	Grade 5	∙ Nature and Effects of Gateway Drugs Caffeine Tobacco Alcohol ∙ Impact of the Use and Abuse of Gateway Drugs Individual Family Community ∙ Prevention and Control of Use and Abuse of Gateway Drugs Development of Life Skills (resistance, decision-making, communication, assertiveness) Observance of Policies and Laws such as school policies and national law (RA 9211 or the Tobacco Regulation Act of 2003)	Health	
	Grade 5	∙ Pagmamalasakit sa kapwa (Concern for others) ∙ Pagkamahabagin (Compassion) ∙ Pagkamagalang (Respect)		
	Grade 6	∙ Healthy School and Community Environments including psychosocial environment (warm atmosphere, healthy interpersonal relations, free from abuse and discrimination)	Health	
Junior high school (12)	Grade 7	∙ Holistic Health includes physical, mental/ intellectual, emotional, social, and moral-spiritual;	Health	
	Grade 7	∙ Development of self-awareness and coping skills	Health	
	Grade 7	∙ Nutrition problems of adolescents includes Eating disorders (i.e., Anorexia nervosa, Bulimia, Compulsive eating disorder)	Health	
	Grade 7	∙ Introduction of Mental Health	Health	
	Grade 7	∙ Understanding stress Eustress Distress ∙ Common areas of stressor that affects adolescents (peer, family, school, community) ∙ Coping with stress	Health	
	Grade 7	∙ Coping with dying and death	Health	
	Grade 7	∙ Types and Management of Common Mental Disorders Identifying triggers and warning signs Prevention coping and treatment Mood disorders, bipolar, schizophrenic, Obsessive Compulsive Disorder (OCD), Obsessive Compulsive Personality Disorder) (OCPD), post-traumatic	Health	
	Grade 8	∙ Gateway Drugs Cigarettes Alcohol ∙ Protective and Risk Factors in the Use of Cigarettes and Alcohol ∙ Prevention, and Control of Gateway Drugs	Health	
	Grade 8	∙ Emotion Moral and ethical values Religious studies (Love of God) Social values (Responsibilities to family, the environment, and the country)	Edukasyon sa Pagpapakatao (Values Education)	
		∙ Mga Karahasan sa Paaralan (Violence in School)		
	Grade 9	∙ Drug Scenario in the Philippines	Health	
	Grade 9	∙ Factors that influence substance use and abuse ∙ Drugs/substances of abuse Stimulants Depressants Narcotics Hallucinogen Inhalants ∙ Harmful effects of drugs on the body Short-term Long-term	Health	
	Grade 9	∙ Types of intentional injuries Bullying (cyber bullying) Stalking Extortion Gang and youth violence Illegal fraternity-related violence Kidnapping and abduction Acts of terror Domestic violence Suicide Sexual victimization and other forms of sexual abuse and harassment ∙ Prevention and management of intentional injuries Self-protection Preventing self-harm Promoting a culture of non-violence through healthful behaviors Reporting cases of violence to proper authorities Seeking help from trusted individuals and health professionals	Health	
High school (4)	Grade 10	∙ Existing National Laws Related to Health Trends, Issues, and Concerns (including) Tobacco Regulation Act of 2003 (RA 9211) Comprehensive Dangerous Drugs Act of 2002 (RA 9165)	Health	
	Grade 10	∙ Paggalang sa Buhay (Respect for Life)	Edukasyon sa Pagpapakatao (Values Education)	
	Grade 10	∙ Paninindigan Tungkol sa Pangangalaga ng Sarili Laban sa Pang-aabusong Sekswal Tungo sa Maayos na Pagtingin sa Sarili at Pagtataguyod ng Dignidad ng Tao (A Stand on Protecting oneself Against Sexual Abuse Towards a Healthy Self-Esteem and Promotion of Human Dignity (includes child sexual abuse, child protection, human trafficking)	Edukasyon sa Pagpapakatao (Values Education)	
	Grade 11,12	∙ Coping with Stress in Middle and Late Adolescence ∙ Mental Health and Well-being in Middle and Late adolescence ∙ Emotional Intelligence ∙ Personal Relationships	Personal Development

**Table 10:** Indonesia national curriculum mapped on school-based mental health literacy

School category (total number of curricula)	Grade	Contents	Subject	Reference number/URL
Primary schools (3)	1,2,3,4,5	Self-awareness: self-observation, self-understanding, Self-uniqueness	Religious education	https://www.datadikdasmen.com/2019/06/unduh-dokumen-kurikulum-2013-sekolah.html
	3,5	Implementation of religious beliefs in managing emotions	Religious education	
	2,3	Management of conflict: Implementation of religious beliefs by developing good behaviors in face of a conflict as a preventive measure for bullying	Religious education	
Middle schools (2)	7,8,9	Having sense of responsibility, care, confidence; Developing a healthy lifestyle; Understanding the responsibility of being a part of a community	Social studies, religious education	
	7	Understanding the concept of association between degree of physical fitness and overall health (including mental health)	Physical education	
Senior high schools (3)	10,12	Understanding character and self-control	Religious education	
	10	Understanding characteristic of mature adults including the emotional aspects	Religious education	
	11	Understanding suicide	Religious education

**Table 11:** Japan national curriculum mapped on school-based mental health literacy

School category (total number of curricula)	Grade	Contents	Subject	Database	Reference number/URL
Elementary school (5)	3	Healthy living ∙ Healthy living ∙ How to live a day in the life ∙ Physical hygiene ∙ Environment of a surrounding area	Health and Physical Education	Guideline of health education for elementary school, 2019 (Ministry of Education, Culture, Sports, Science and Technology)	https://www.mext.go.jp/a_menu/kenko/hoken/__icsFiles/afieldfile/2019/07/12/1334052_2.pdf
	4	Physical growth and development ∙ Physical growth and development ∙ Changes in the body during adolescence ∙ Life for better growth and development of the body	Health and Physical Education		
	5	Mental health ∙ Development of the mind ∙ Close relationship between mind and body ∙ Coping with anxiety and worries	Health and Physical Education		
	5	Injury prevention ∙ Occurrence of injury ∙ Preventing injuries caused by traffic accidents ∙ Prevention of injury and criminal damage caused by daily life ∙ Treatment of injuries	Health and Physical Education		
	6	Disease prevention ∙ How diseases occur ∙ Prevention of diseases caused mainly by pathogens ∙ Prevention of diseases caused primarily by lifestyle behaviors ∙ Smoking, drinking alcohol, drug abuse and health ∙ Various community health initiatives	Health and Physical Education		
Junior high school (7)	7	Origin of health and causes of disease/lifestyle and health ∙ Origin of health and causes of disease/Lifestyle and health ∙ Exercise and health ∙ Diet and health ∙ Rest, sleep, and health/Harmonious living	Health and Physical Education	Guideline of health education for junior high school students, 2020. (Ministry of Education, Culture, Sports, Science and Technology)	https://www.mext.go.jp/a_menu/kenko/hoken/__icsFiles/afieldfile/2020/20200317-mxt_kensyoku-01.pdf
	7	Coping with cravings and stress and mental health ∙ Mental and physical relationships ∙ Coping with cravings ∙ Coping with stress	Health and Physical Education		
	8	Prevention of lifestyle-related and other diseases ∙ Causes of lifestyle-related diseases ∙ Prevention of lifestyle-related diseases ∙ Mechanisms and causes of cancer ∙ Prevention and early detection of cancer	Health and Physical Education		
	8	First aid ∙ Significance of first aid ∙ Methods and practice of first aid ∙ Practice of first aid	Health and Physical Education		
	8	Aiming for behavior that respects the personality of self and others—Thinking about sexual problems ∙ Resolving adolescent anxieties and worries and coping with sexual development ∙ Forming healthy and safe attitudes and habits, both mentally and physically	[Special activity] Health and Physical Education		
	9	Prevention of infectious diseases ∙ Infectious diseases ∙ Prevention of infectious diseases ∙ Prevention of sexually transmitted diseases ∙ Prevention of AIDS	Health and Physical Education		
	9	Health and environment ∙ Ability to adapt to the environment ∙ Environment related to the activity ∙ Hygienic management of indoor air ∙ Hygienic management of drinking water ∙ Hygienic management of garbage and other waste ∙ Learning issues related to health and environment	Health and Physical Education		
High school (9)	10	Health concepts ∙ National health challenges ∙ Concept and origin of health ∙ Appropriate decision-making and behavioral choices and environment for the maintenance and promotion of health	Health and Physical Education	Guideline of health education for high school students, 2021 (Ministry of Education, Culture, Sports, Science and Technology)	https://www.mext.go.jp/a_menu/kenko/hoken/20210310-mxt_kouhou02-1.pdf
	10	Modern infectious diseases and their prevention ∙ Modern infectious diseases ∙ Prevention of infectious diseases ∙ AIDS and sexually transmitted diseases	Health and Physical Education		
	10	Prevention and recovery from lifestyle-related diseases ∙ Prevention and recovery from lifestyle-related diseases ∙ Prevention of cancer ∙ Cancer prevention and recovery	Health and Physical Education		
	10	Smoking, drinking alcohol, drug abuse and health ∙ Smoking, drinking alcohol and health ∙ Drug abuse and health	Health and Physical Education		
	10	Prevention and recovery from mental illness Characteristics of mental illness ∙ Coping with mental illness	Health and Physical Education		
	10	First aid ∙ Significance of first aid ∙ Cardiopulmonary resuscitation ∙ Routine first aid	Health and Physical Education		
	11	Health throughout each lifespan ∙ Adolescence and health ∙ Marriage and health ∙ Aging and health	Health and Physical Education		
	11	Labor and health ∙ Workplace accidents and occupational diseases ∙ Workplace health and safety management ∙ Maintenance and promotion of workers' health	Health and Physical Education		
	11	Health and medical systems and local health and medical institutions ∙ Health and medical care system in Japan ∙ Utilization of local health and medical institutions ∙ Pharmaceutical system and its utilization	Health and Physical Education		

## Results

This study identified the two main findings from the content analysis of both policies and curriculum on mental health literacy among three countries. First, general mental health education is partially present in two (Philippines and Japan) of the three countries, with the Philippines having a robust health education curriculum. Mental health literacy education has been provided as health and physical education in Japan; it starts in grade 5 and stops in grade 10. In the Philippines, it starts in kindergarten and stops at grade 12 through health, values education, homeroom guidance. In Indonesia, religious education includes mental health literacy as an integrated subject; it starts in grade 1 and stops at grade 12.

Second, mental health literacy is partially or somewhat provided in two of the three countries. This study identified that general mental health education has been implemented according to the policies related to mental health in the Philippines and Indonesia. In Japan, a core policy of mental health literacy has not yet been formulated; however, the curriculum on mental health literacy was developed and implemented according to the course of study.

This three-country analysis revealed a diverse educational contexts as mental health literacy education is related to religious education in Indonesia and the Philippines, but Japan has provided it mainly through health and physical education. Although the three Asian countries have commonalities that were formulating mental health-related legislation, they have diverse backgrounds related to several subjects or educational approaches, therefore this study targeted these countries to identify any commonalities or similarities.

### Policy contents and its actors

This study clarified the policy contents and its actors, showing the characteristics of school mental health policy formulation in three Asian countries (Table 6).

The Philippines showcases a holistic approach that includes systematic, scaffolded, developmentally appropriate, and comprehensive across all ages to mental health in schools. Holistic aspects are focused on the community-based approach, this is emphasized that local and religious leaders play an important role in mental health care, and families and the whole society are encouraged to support patients. National policies are in place for the implementation of mental health programs. Broader health policies such as the Universal Health Care Act and the Guidelines on the Healthy Settings Framework in Learning Institutions include the need to integrate strategies for mental health in educational institutions for actual mental health-sustaining strategies. Additionally, mental health strategies are also commonly integrated in educational policies such as the Good Manners and Right Conduct (GMRC) and Values Education Act, and the Health Operation Policy (*Oplan Kalusugan* Policy). The implementation of the GMRC and Values Education Act and the Comprehensive Dangerous Drugs Act of 2002 represents a commitment to integrating mental health into the educational framework. These policies are crucial in setting a foundation for a supportive environment that caters to the mental health needs of students who have any problems with mental disorders.

Indonesia focuses on religious education in primary and secondary education, with specific ministerial regulations, and this showcases a dynamic approach to mental health in schools. Dynamic aspects mean that this requires flexible mental health strategies that can adapt to local challenges and cultural backgrounds. The implementation of the Enhanced Basic Education Act and the GMRC Act represents a commitment to integrating mental health into the educational framework. These policies are crucial in setting a foundation for a supportive environment that caters to the mental health needs of students.

In Japan, the Ministry of Health, Labor and Welfare published the “Act on Mental Health and Welfare for the Mentally Disabled” in 1950, which obligates the public to maintain and promote mental health, deepen their understanding of persons with mental disorders, and cooperate with the efforts of persons with mental disorders to overcome their disabilities, return to society, and participate in social and economic activities. However, its implementation has not yet been evaluated such as their perception or knowledge that the regulation could contribute to enhancing the ability and awareness among them. In 1958, both the Ministry of Education, Culture, Sports, Science and Technology (MEXT) and the Ministry of Health, Labor and Welfare enacted the “School Health and Safety Act” and its enforcement regulations, which contains detailed regulations on health examinations and mental health support. This act provides a structured and comprehensive approach for addressing students’ mental health through local medical institutions and by providing health counseling by *Yogo* Teacher (school nurse). In addition, the MEXT revised the act in 2017, which promotes the establishment of school counselors and school social workers to support students’ mental health and welfare.

### Policy mapping for the evaluation of school-based mental health policies

Table 7 shows the summary of the evaluation of the national policies in the three Asian countries according to the key points of global UN agencies’ mental health guidelines and manuals [9]. There appear to be specific commonalities among the three Asian countries in the policies that were reviewed, these are purpose, scope of mental health issues addressed, and dissemination. In assessing the purpose among three countries, it was presumed that school mental health policies had been clearly defined and accurately addressed the challenges of mental illness and health concerns faced by children and adolescents in each country.

This study identified several implementers engaged in school-based mental health literacy which are described in Table 6. On the other hand, there is a difference in the role of schools for mental health literacy. In Japan, multiple implementers who are in charge of *Yogo* Teacher (school nurse) or school counselors, medical institutions, national and local governments, then they engage to support mental health among students. In the Philippines, school-based mental health literacy education was implemented by several implementers such as the Philippine Council for Mental Health, Department of Social Welfare and Development (DSWD), and National Center for Mental Health (NCMH). In Indonesia, the efforts have included schools, educational institutions, the Ministry of Religious Affairs, religious leaders, and community representatives.

### Curriculum implementation of school-based mental health literacy education

The school-based mental health literacy, and how each national curricula in three Asian countries were summarized, and the development of the national curricula is described in Table 8. The curriculum analysis identified a specific challenge: a lack of “*first aid skills to support others who are developing a mental disorder or are in a mental health crisis*”, this would involve the extracted actors to support them.

In the Philippines, mental health literacy is taught in subjects such as health, values education, and homeroom guidance. On the other hand, in Japan, almost all mental health literacy is taught through health and physical education subjects. In terms of curriculum content, both the Philippines and Japan include stress management, social and emotional learning and prevention of smoking, drinking, and drug abuse to prevent substance issues. In contrast, Indonesia includes mental health education in religious education. Similar to the Philippines’ subject on values education, comprehensive mental health education has been provided throughout religious education, such as self-awareness: self-observation, self-understanding, and self- awareness from primary school. In both countries, topics such as dealing with emotions, suicide, and prevention of bullying are also discussed. All three countries emphasize the development of both physical and mental health, with Indonesia using the term comprehensive health and the Philippines using the term holistic approach.

In Japan, the course of study (guideline of health education) published by MEXT included detailed content on mental health and illness, focusing on prevention, recovery, and coping strategies, indicating an emphasis on resilience and mental strength. In the Philippines, mental health literacy education was integrated into the K-12 curriculum with the goal of making learning competencies age-appropriate and properly sequenced in terms of progression. In Indonesia, the mental health literacy curriculum is absent in the curriculum and policy in Usaha Kesehatan Sekolah (UKS), which is the school health policy; mental illness and how to seek support are not specified in the documents. Religious education includes broad topics such as self-awareness, emotional management, and conflict resolution, which are reflected in moral education and character education, and it was indicated that mental health literacy is not yet a part of the health curriculum in Indonesia. Therefore, this shows that religious education plays a significant role in providing mental health literacy education, including specific policies related to mental health.

## Discussion

This study identified that mental health education is partially implemented in the Philippines, Indonesia, and Japan. The Philippines provides a comprehensive curriculum from kindergarten to 12, while Japan includes it in health education from grades 5 to 10. In Indonesia, mental health literacy is integrated into religious education from grades 1 to 12. While the Philippines and Indonesia align their efforts with mental health-related policies, Japan lacks a core policy but incorporates mental health literacy through its national curriculum. This study provides an overview of health education curricula, encompassing mental health education, social and emotional learning, as well as religious and character education. It highlights how the authors—experts in their respective fields—adopt a broad interpretation of mental health literacy in their analysis.

As shown in both the policy content analysis (Table 6) and the policy mapping analysis (Table 7), all three countries demonstrate the involvement of various professionals in delivering school mental health services, including relevant ministries, school counselors, and local governments. In the Philippines, the approach to personnel in educational institutions is particularly notable for its community-centered and multidisciplinary focus. This strategy emphasizes the importance of engaging a broad range of stakeholders, including educators, guidance counselors, and community members, to promote mental health within educational settings. In Indonesia, a key feature of the approach is the collaboration among professionals from diverse disciplines. This network ensures that school mental health services are comprehensive, involving doctors, nurses, midwives, and local community leaders, such as religious figures and school cadres (including student empowerment initiatives). In Japan, the utilization of specialized personnel like *Yogo* Teacher (school nurse), school counselors, and school social workers highlights the country’s dedication to providing expert mental health care and support. Japanese schools employ school counselors as a psychologist to provide an individual consultation with students or parents for prevention of bullying or school absenteeism. School counselors in Japan are professionals who support the mental health of students, and they use their specialized knowledge, primarily in psychology, to deal with a variety of issues within schools such as providing counseling and preventive education.

In the case of Japan, in April 2023, the Japanese government launched the Children and Family Agency to advance evidence-based policymaking (EBPM) which aims to address child poverty and mental health among children and adolescents [33]. The pandemic’s impact has reached far beyond academic disruptions, leading to increased psychological stress caused by prolonged school closures, social isolation, and the widespread uncertainty of the situation [34]. A survey in Japan found that the monthly incidence of eating disorders in children rose during the COVID-19 pandemic period [35]. This public health crisis has influenced an urgent need to reevaluate, redefine, and enhance mental health strategies in schools, making it a crucial aspect of an appropriate public health response [36]. A 2023 systematic review by Baffsky et al. identified several strategies with high potential for effective implementation of school mental health prevention programs. These strategies include conducting audits and providing feedback, engaging principals as key local leaders, increasing teachers’ commitment, and scheduling regular meetings for school team programs [37]. These pressing issues underscore the need for the development and implementation of comprehensive policies.

The Japanese New Center for Health Control (JNCHC) should disseminate evidence-based mental health information for children not only during pandemics but also in other public health crises that affect society [38]. This is because early intervention, using NCGM’s services, is cost-effective, and the center should address mental health issues among children and adolescents such as suicide, refusal to attend school, eating disorders, and social media use during times of outbreaks. To address these issues, the JNCHC should develop culturally relevant recommendations and work with the Child and Family Agency and the Ministry of Health. For example, this kind of collaboration is already taking place in Ichikawa City, Chiba Prefecture, Japan, where a regional network combines healthcare, education, and justice services to assist children and adolescents with mental disorders. This network utilizes maternal and child health facilities as safety nets at the local level [39].

The experiences of the Philippines, Indonesia, and Japan showcase various strategies for incorporating mental health into educational systems, however, this should acknowledge that it is not always specific to mental health literacy. Despite differences in culture and context, all three countries stress the need for a multifaceted approach that includes policy development, curriculum integration, comprehensive support services, community involvement, and specialized resources. These approaches offer valuable insights for improving school mental health, especially in the face of challenges like the COVID-19 pandemic. This comparative analysis underscores the complexity of addressing student mental health needs and highlights the importance of integrating mental health into educational systems for future initiatives. These approaches offer insights into improving school mental health. For example, Japan integrates mental health into health education, the Philippines includes it in health, values education and homeroom guidance, and Indonesia incorporates it into religious education. This highlights the importance of embedding mental health into educational systems for future initiatives.

The analysis of curricula on mental health literacy as well as its construction in the three countries showed the characteristics and specific subjects of the curricula. In the Philippines, the multifaceted approach to school mental health, which includes policy development, curriculum integration, and comprehensive support services, exemplifies a holistic and inclusive strategy. This approach emphasizes the creation of nurturing policies that embed mental health education and support into the school environment. In Indonesia, it focuses on fostering a healthy school environment and developing foundational emotional and social skills, which represents a dynamic and preventive strategy. “A dynamic and preventive strategy” means that the approach in Indonesia emphasizes anticipating and addressing mental health needs early by fostering a positive school environment and teaching essential emotional and social skills. This strategy aims to prevent mental health issues from developing rather than waiting to intervene after problems emerge. This method highlights the importance of early intervention and creating a supportive atmosphere that promotes mental well-being from a young age, equipping students to handle mental health challenges effectively. In Japan, the structured and comprehensive approach, which includes a curriculum centered on prevention, recovery, and coping strategies, along with the allocation of specialized personnel, reflects a strong commitment to providing expert care and support. This strategy underscores the necessity of a well-resourced framework to address diverse mental health needs in students. The previous study by Ojio et al. in 2021 indicated that while adaptation to school-based mental health literacy may be limited, educator interventions can still significantly benefit students within the school setting [22]. Therefore, interventions should begin earlier, including teacher training to provide mental health literacy education, to better address adolescents at risk of developing mental health issues.

Based on the findings of the study, several country-specific recommendations can be made for enhancing school-based mental health literacy in the three Asian countries. In the Philippines, the implementation of mental health programs should continue to be supported by national policies, with a focus on integrating mental health strategies into broader health and educational policies. For instance, it is suggested to have more comprehensive incorporation of mental health education into the K-12 curriculum, and provision of enhanced school-based mental health services. The holistic approach to mental health in schools should be further strengthened. In Indonesia, the focus on guidance and counseling in religious education between primary and secondary schools should be further developed, with the integration of mental health education into religious education. Religious education can foster greater self-awareness and a deeper understanding of religious beliefs and practices, as well as their impact on individuals, families, and communities [40]. By prioritizing the effective implementation of religious education, school-based mental health education and promotion strategies can amplify its positive effects, contributing to improved adolescent mental health.

In Japan, a specific policy for school-based mental health literacy should be formulated to provide a structured and comprehensive approach to addressing students’ mental health needs. The detailed curriculum on mental health literacy should also be further developed systematically and implemented according to the course of study. Moreover, the common recommendations among three Asian countries include monitoring and evaluation of policies on school-based mental health literacy. Collaboration with UN agencies should be considered to align national approaches with international recommendations. Tailored approaches that address the diverse cultural and educational contexts of each country should be developed and implemented. This study recommended target frameworks or guidelines by the UN agencies that constructed the concept of health-promoting schools since school-based mental health should be strengthened throughout the comprehensive approach [10].

In summary, the recommendations for enhancing school-based mental health literacy in the three countries focus on improving student outcomes, teacher preparedness, and policy implementation. By integrating mental health education into national curricula and policies, students will benefit from a structured and supportive learning environment. Teacher preparedness will be strengthened through comprehensive training and guidance on addressing mental health issues in the classroom. Additionally, clear and consistent policies will ensure effective implementation and monitoring, leading to sustainable progress in mental health literacy education.

## Limitations of the study

This study has several limitations. First, it did not include interviews with policy implementers and relied on retrospective analysis of data sources. An evaluation of the actual implementation of both policies and basic education curricula on mental health literacy is beyond the scope of this study. Second, the study did not investigate the health and education outcomes (epidemiological data) following school mental health interventions. Due to the lack of epidemiological data, no outcomes that might impact policy and curriculum implementation could be considered. Third, the study focused on the Philippines, Indonesia, and Japan, and its findings may not be applicable to other Asian countries due to differences in population demographics and socio-cultural backgrounds (Table 1). Fourth, mental health educational interventions by the private sector were not part of the study and therefore any roles and contributions of the private education sector on mental health literacy and services have not been reflected in this study. Additionally, further research is needed on the implementation and evaluation of school-based mental health literacy, including assessments of its impact on educators.

## Conclusion

This study highlighted the partial implementation of mental health literacy education in the Philippines, Indonesia, and Japan. The Philippines offers a comprehensive curriculum (Kindergarten to Grade 12), Japan includes it in health education (grades 5–10), and Indonesia integrates it into religious education (grades 1–12). While the Philippines and Indonesia align with mental health policies, Japan addresses mental health literacy through its national curriculum without a core policy. The study underscores a broad interpretation of mental health literacy across health, social, emotional, and character education. The curriculum analysis identified a key challenge: the lack of “*first aid skills to support others who are developing a mental disorder or experiencing a mental health crisis*”, highlighting the need for trained actors (e.g., teacher, counselor) to provide such support. Common recommendations for the three Asian countries include monitoring and evaluating school-based mental health policies, collaborating with UN agencies to align national approaches with international standards, and developing tailored strategies that reflect each country’s unique cultural and educational contexts.

## Data Availability

No datasets were generated or analyzed during the current study.

## Abbreviations

CDC: Centers for Disease Control and Prevention
COVID-19: Coronavirus disease 2019
DOE: Department of Education
GMRC: Good manners and right conduct
HPS: Health promoting school
MEXT: Ministry of Education, Culture, Sports, Science and Technology
MOE: Ministry of Education
MOH: Ministry of Health
NCGM: National Center for Global Health and Medicine
NDOE: National Department of Education
STEAM: Science, technology, engineering, arts, and mathematics
UN: United Nations
WHO: World Health Organization
